# Therapeutic massage with nanostructured lipid carrier gel containing quercetin for muscle injury treatment in rats

**DOI:** 10.1590/1414-431X2025e14924

**Published:** 2026-01-09

**Authors:** J.M. Bellé, J.P. de Moraes, R.P. Martins, G.O. Puntel, C. Franco, V.C. Rech, L.U. Signori

**Affiliations:** 1 Programa de Pós-Graduação em Reabilitação Funcional, Departamento de Fisioterapia e Reabilitação, Universidade Federal de Santa Maria, Santa Maria, RS, Brasil; 2 Programa de Pós-Graduação em Nanociências, Universidade Franciscana, Santa Maria, RS, Brasil

**Keywords:** Inflammation, Oxidative stress, Rehabilitation, Solid lipid nanoparticles (SLN), Skeletal muscle, Massage

## Abstract

Quercetin has therapeutic potential in the treatment of musculoskeletal lesions, but presents poor oral absorption because of its low water solubility and structural instability. Its penetration through the skin can be enhanced by quercetin-loaded nanostructured lipid carriers (NLC-Q), which is increased when applied with massage, but this has not yet been tested. This study aimed to evaluate the effect of massage with NLC-Q gel on biochemical parameters after a traumatic lesion of the gastrocnemius muscle. Forty-five male Wistar rats were divided into five groups (control, lesion, lesion treated with NLC-Q gel, lesion treated by massage with placebo gel, and lesion treated by massage with NLC-Q gel). The gastrocnemius muscle was lesioned by mechanical crushing, and treatments began 24 h after injury. Massage was performed at 12 h intervals for a total of five 5-min sessions. Serum and muscle creatine kinase (CK) concentrations and muscle oxidative stress (concentration of reactive oxygen species [ROS], lipid peroxidation, protein carbonyls, and activities of superoxide dismutase and catalase enzymes) were evaluated 96 h after lesion formation. Blood CK levels increased in all injured groups (P<0.001); however, the interventions reduced plasma CK compared to the lesion group (P<0.05). Interventions reduced lipid peroxidation (P<0.05), but only the NLC-Q gel and massage with NLC-Q gel reduced the concentration of ROS and protein oxidation in the lesion group (P<0.05). These findings indicated that NLC-Q gel and massage with NLC-Q gel can help repair muscle damage and reduce oxidative stress parameters.

## Introduction

Traumatic musculoskeletal injuries can be either indirect (strains or tears) or direct (contusions or lacerations) and can result in functional deficits (1,2). These injuries account for approximately 90% of sports injuries (2) and can interfere with physical activity, ultimately affecting overall health status (3). Contusions are the most common sports injuries, and degeneration/inflammation occurs within the first few minutes after injury and can last for two weeks (4). During this phase, the myofibrils are destroyed (1,2) through chemotactic signals that stimulate the infiltration of blood inflammatory cells (neutrophils and monocytes), thereby reinforcing the action of the macrophages present in the tissue in phagocytizing damaged cells (1).

Oxidative stress (OS) is at the heart of the cellular signaling in musculoskeletal injuries, including an imbalance between the production of reactive oxygen and nitrogen species (RONS) and the ability of cells to neutralize them through antioxidant defenses (5-9). Under these conditions, OS is a physiological process that is involved in tissue repair. However, excess production of pro-oxidants can alter the molecular functions of cells directly (lipoperoxidation and damage to proteins and DNA) and/or indirectly (leukocyte activation) (5), thus causing additional damage to myofibrils by amplifying the inflammatory response (10).

Different therapeutic strategies that attenuate excess RONS production have been used to reduce secondary muscle damage resulting from excessive stimulation of pro-inflammatory pathways, thereby promoting recovery and functionality (6,7,9,11). Thus, exogenous non-enzymatic antioxidants, such as quercetin, have a favorable action in restoring redox balance (9,12). Quercetin (C_15_H_10_O_7_) is a dietary polyphenol that belongs to the flavonoid class, exhibiting antioxidant, anti-inflammatory, antiproliferative, anticancer, antidiabetic, and antiviral properties (12,13). However, its low water solubility, structural instability, and extensive first-pass metabolism reduce its absorption and bioavailability, resulting in low systemic levels of free quercetin following oral administration (14). Therefore, dermal application has emerged as an alternative route of delivery.

Several methods of dermal and transdermal drug delivery based on carrier nanotechnology are used to overcome the skin permeability barrier and improve the bioavailability of the active ingredient (15). The use of lipid-based drug carriers can overcome the low bioavailability and systemic stability of quercetin (13). Nanostructured lipid carriers are composed of solid and liquid lipids that form imperfect crystalline structures. These imperfect structures support greater drug loading with greater retention efficiency and physical stability during storage, which increases solubility, controls the release rate, and improves the bioavailability of encapsulated lipophilic compounds (16). Nanocarriers can be applied synergistically with other physical methods to improve transdermal absorption and increase drug penetration (17).

Therapeutic massage is widely used in sports and can improve psychological aspects and reduce pain resulting from high-intensity exercise (18). Additionally, this approach can mitigate OS (19). However, manual massage therapy requires a slippery substance (e.g., oil, cream, or gel) to reduce the friction between the therapist's hands and the patient's skin. The penetration and effectiveness of a gel containing nanostructured lipid carrier loaded with quercetin (NLC-Q) (9,20) can be enhanced through massage therapy, and these therapies can act synergistically in the recovery of musculoskeletal injuries. However, this combination has not yet been tested. The combination of massage with an NLC-Q gel represents a promising approach for greater treatment efficacy in musculoskeletal injury. This study aimed to evaluate the effects of therapeutic massage with NLC-Q gel on biochemical parameters after traumatic lesions to the gastrocnemius muscle in rats.

## Material and Methods

### Experimental design

Animal handling was carried out in accordance with the appropriate animal testing guide (according to Brazilian Legislation No. 11,794/2008). All procedures described in this study were approved by the Ethics Committee on Animal Use of the Franciscan University (No. 02/2019).

Forty-five male *Rattus norvegicus Albinus* were obtained from the Central Animal Facility of the Federal University of Rio Grande do Sul (UFRGS). All rats were approximately 90 days old and weighed between 250 and 300 g. During the study, the animals were kept in a room with a 12-h light/dark cycle, which was maintained at an average temperature of 22±2°C. The animals were housed in collective cages (3 per cage) with access to drinking water and a standard commercial diet *ad libitum*.

After the injury protocol, the animals were randomly divided using a computer program (www.random.org) into five homogeneous groups of nine animals each: control, lesion, NLC-Q gel, massage with placebo gel, and massage with NLC-Q gel.

### Gel containing a nanostructured lipid carrier loaded with quercetin (NLC-Q)

NLC-Q was developed according to previous studies (9,20). The high shear rate method utilized an Ultraturrax^®^ (T-18, IKA^®^, Brazil) to produce the NLC-Q. One hundred milliliters of NLC-Q were produced according to the composition described in the organic phase (Inwitor 900K: 0.8 g [IOI Oleo GmbH, Germany]; Crodamol^®^: 4.2 g [IPP Pharma, Brazil]; Span 60: 1.0 g [ZTCC, China]; Quercetin: 0.1 g [YTBIO, China]) and aqueous phase (Tween 80: 2.0 g [INLAB, Brazil]; MilliQ Water: 92 mL). Quercetin was then added to the organic phase and stirred for an additional 5 min. Subsequently, the aqueous phase was added to the organic phase and stirred for 10 min. The NLC-Q was prepared at room temperature at 2,354 *g* using an Ultraturrax^®^ (T-18, IKA^®^) and then packaged in 100-mL amber flasks. After preparation, three NLC-Q gel samples (8 g) were placed in test tubes and centrifuged (model TDL 80-2B, China) at room temperature for 30 min at 1,000 *g*.

Then, a 100-mL gel with NLC-Q was prepared using the dispersion method. Initially, 0.4 g of Carbopol ETD 2020^®^ (Lubrizol, Brazil) (0.4%), 0.3 g of Germall (115 Ashland, Brazil) (0.3%), and 0.25 g of Triethanolamine (Adcos Professional, Brazil) (0.25%) were weighed. These substances were individually mixed manually using a pistil in a porcelain crucible in the following order: Carbopol, Germall, and the Triethanolamine emollient solution. Subsequently, 99.05 mL of NLC-Q was added to the mixture, which was manually stirred with the pistil until it was fully homogenized. After preparation, three NLC-Q gel samples (8 g) were placed in test tubes and centrifuged (centrifuge model TDL 80-2B, China) at room temperature at 1000 *g* for 30 min. This test causes an increase in gravity, enhancing the mobility of the particles, which can lead to phase separation, sediment or supernatant formation, and coalescence. Any sign of instability indicates that the product must be reformulated (9,20).

### Muscular lesion

Muscle injury was induced noninvasively in the gastrocnemius muscle (right) according to a specific methodology for muscle injury in rats (6), with a few modifications (8). Before injury, the animals were anesthetized by intraperitoneal administration of ketamine hydrochloride (50 mg/kg) and xylazine hydrochloride (10 mg/kg). After anesthesia, all animals were depilated, and the injury protocol was carried out. The animals were positioned in ventral decubitus on the base of the injury generator equipment, with the knee fully extended and the ankle in a neutral position (90°). A 200-g mass was dropped through a polyvinyl chloride tube (used as a guide), which was 30 cm in height and 20 mm in diameter, placed directly onto the right gastrocnemius muscle venter, generating an impact force of 0.484 N. Two impacts were performed. The rats in the control group were anesthetized to ensure standardization, but did not receive muscle trauma.

### Interventions

**Figure 1:**
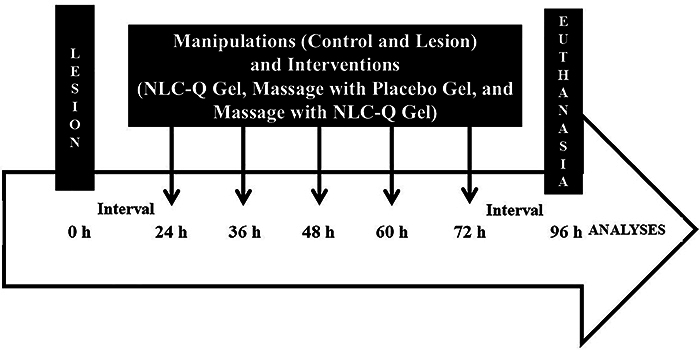
Study design. h: hour; NLC-Q Gel: gel with nanostructured lipid carriers with quercetin.

Twenty-four hours after the lesion, the interventions began, which occurred every 12 h (5 interventions: 24, 36, 48, 60, and 72 h) until 72 h, and euthanasia was carried out after 24 h (96 h). The study design is illustrated in Figure 1.

Control Group: the animals were handled but not injured. Lesion Group: the animals were injured and manipulated, but received no treatment. NLC-Q Gel Group: NLC-Q gel was spread on the injured area and left for 5 min. Massage with Placebo Gel Group: an inert gel (commercial ultrasound gel) was spread on the local lesion, and therapeutic massage was performed. Massage with NLC-Q Gel Group: NLC-Q gel was applied to the injured area, and then therapeutic massage was performed. Massage was performed at 12-h intervals for a total of five sessions lasting 5 min each. An equal volume of gel was applied to the injury site in each group. One experienced researcher performed bare-finger massages (21). The protocol consisted of scaled-down versions of several common modalities. These included (in order) gentle mobilization, skin rolling, and deep strokes (also known as “muscle stripping” or “myofascial release”) to the injury site (22). The animals were mechanically prevented from bringing their mouths to the injury site during the procedure. At the end of the procedure, the injury site was washed with water to remove any remaining gel.

### Euthanasia protocol and sample preparation for biochemical analysis

After the experimental period (96 h), the rats were euthanized by intraperitoneal anesthesia with an overdose of ketamine (500 mg/kg). Whole blood was collected in heparinized tubes by intracardiac puncture. Blood samples were cooled (5°C) for subsequent creatine kinase (CK), electrolyte, erythrogram, and leukogram analyses. The right gastrocnemius muscle was removed and divided into two parts. One part was quickly homogenized in saline solution (150 mM) and stored on ice. After homogenization, the skeletal muscle samples were frozen (-80°C) to be used to determine biochemical markers. Dissection of the gastrocnemius muscles did not reveal signs of bone fracture in the analyzed area.

### Biochemical analyses

Erythrogram and leukogram measurements were performed using a veterinary hematology analyzer (VET Mindray BC-2800, China). The pH was evaluated using a biochemical analyzer (Mindray IN BS-200, China). Electrolytes (potassium, sodium, chloride, and calcium) were analyzed by a selective ion electrode module (WAMA Diagnostics WE-300, China).

CK concentration was measured based on the kinetic activity of the enzyme in blood plasma samples as an index of muscle contusion injury using diagnostic kits (CK-NAC Liquiform, Labtest, Brazil) on a biochemical analyzer (Mindray BS-200). The activity of the CK enzyme in the gastrocnemius muscle was evaluated using a colorimetric method. After incubation for 20 min at 37°C in a water bath (DeLeo, Brazil) for color development, absorbance was determined using a Synergy HTX spectrophotometer (Biotek, USA) at a wavelength of 540 ηm. The results are reported as µmol of creatine formed per min per mg of protein.

### Oxidative stress markers

Measures of OS in the injured muscle were evaluated. The measures of damage were the total production of reactive oxygen species (ROS), lipid peroxidation (TBARS), and protein oxidation (carbonyls). The antioxidant analysis was assessed by the activities of the enzymes superoxide dismutase (SOD) and catalase (CAT).

ROS production in the muscle homogenates was determined by the oxidation of reduced dichlorofluorescein (H_2_DCF-DA). Briefly, muscle homogenates were added to a standard medium containing Tris-HCl as a buffer (10 mM, pH 7.4) and H_2_DCF-DA (1 mM) for 60 min under lightless conditions. Fluorescence was quantified at 488 Zm for excitation and at 525 Zm for emission, with slit widths of 3 Zm, using a spectrofluorometer (RF-5301 PC; Shimadzu, Japan) with oxidized dichlorofluorescein (DCF) as the standard (23).

Levels of thiobarbituric acid reactive substances (TBARS), primarily malondialdehyde (MDA), were determined as an index of tissue lipid peroxidation, according to the method described by Ohkawa et al. (24). Aliquots of 200 μL of skeletal muscle S1 were added to the color reaction. TBARS levels were measured at 532 ηm using a standard curve of MDA and corrected according to protein content.

The determination of carbonyl was based on the reaction of carbonyl groups with 2,4-dinitrophenylhydrazine (DNPH) to form dinitrophenylhydrazone, a yellow compound, which was measured using a spectrophotometer (Biotek Synergy HTX, USA) at 370 ηm (25). The results are reported as ηmol of carbonyls/mg of protein.

The activity of cytosolic superoxide dismutase (Cu/Zn SOD) was measured in the injured gastrocnemius muscle. Different samples of skeletal muscle (10 to 50 μL) were added to a medium containing glycine buffer (50 mM, pH=10.5) and adrenaline (1 mM). SOD kinetic analysis was initiated after adrenaline addition, and the color reaction was measured at 480 ^η^m (26). SOD enzyme activity is reported in units of enzyme activity per mg of protein.

CAT enzyme activity was measured in the skeletal muscle. Skeletal muscle samples (50 mL) were added to a medium containing potassium phosphate buffer (TFK 50 mM, pH=7.4) and H_2_O_2_ (1 mM). CAT kinetic analysis was initiated after the addition of H_2_O_2_. The color reaction was measured at 240 ηm (27). Protein content was measured using bovine serum albumin (BSA) as the standard, as described in a previous study (8).

### Statistical analysis

Data are reported as means±SD. The distribution of variables was assessed using the Shapiro-Wilk normality test. A one-way ANOVA was conducted to compare data with symmetrical distribution, followed by Tukey's *post hoc* test. The between-group differences are presented as the mean difference (MD), 95% confidence interval (95%CI), and respective percentages. The significance level was set at 5% (P<0.05).

## Results

### Hemogram

The pH, electrolytes, erythrocytes, and platelets showed no between-group differences. Total leukocytes and their fractions also showed no between-group differences at the end of the experiment. Table 1 presents the pH, electrolyte, and hemogram results.

**Table 1:** Results of pH, electrolytes, erythrogram, and leukogram of the different study groups.

Variables	Groups					P-Value
	Control	Lesion	NLC-Q Gel	Massage with Placebo Gel	Massage with NLC-Q Gel	
pH	7.79±0.1	7.85±0.2	7.87±0.3	7.73±0.2	7.63±0.16	0.356
Ca^++^ (mEq/L)	1.49±0.2	1.70±0.1	1.51±1.6	1.74±0.3	1.63±0.4	0.524
Cl^-^ (mEq/L)	105±1.6	105±1.8	105±1.4	106±1.6	106±2.2	0.401
Na^++^ (mEq/L)	137±2.2	137±1.82	131±1.7	136±2.2	135±2.7	0.530
K^+^ (mEq/L)	4.4±0.5	4.1±0.4	4.3±0.4	4.2±0.7	4.1±0.6	0.442
Erythrocytes (×10^5^/mm^3^)	8.33±0.87	8.29±0.46	8.51±0.65	8.44±0.43	8.57±0.20	0.984
Hb (g/dL)	14.5±1.5	14.6±0.8	14.8±1	14.5±0.8	14.8±0.5	0.967
Hematocrit (%)	46.3±5.9	46.96±3.2	46.15±2.3	45.6±1.9	45.7±1.8	0.946
Platelets (×10^3^/mm^3^)	941±115	1062±88	1060±101	1019±160	1108±146	0.134
Total leukocytes (×10^3^/mm^3^)	6257±4064	6457±1510	6300±1781	6171±1481	6314±996	0.999
Neutrophils (×10^3^/mm^3^)	1092±747	884±285	1082±777	692±368	741±254	0.510
Monocytes (×10^3^/mm^3^)	101±86	159±148	127±36	70±13	120±52	0.635
Eosinophils (×10^3^/mm^3^)	2±6	60±90	53±71	17±30	9±22	0.180
Lymphocytes (×10^3^/mm^3^)	5060±3413	5444±1279	5092±1585	5426±1214	5462±774	0.987

Data are reported as means±SD (ANOVA). pH: Hydrogen potential; Ca^++^: Calcium; Cl^-^: Chlorine; Na^++^: Sodium; K^+^: Potassium; Hb: Hemoglobin.

### Serum and muscle creatine kinase

**Figure 2:**
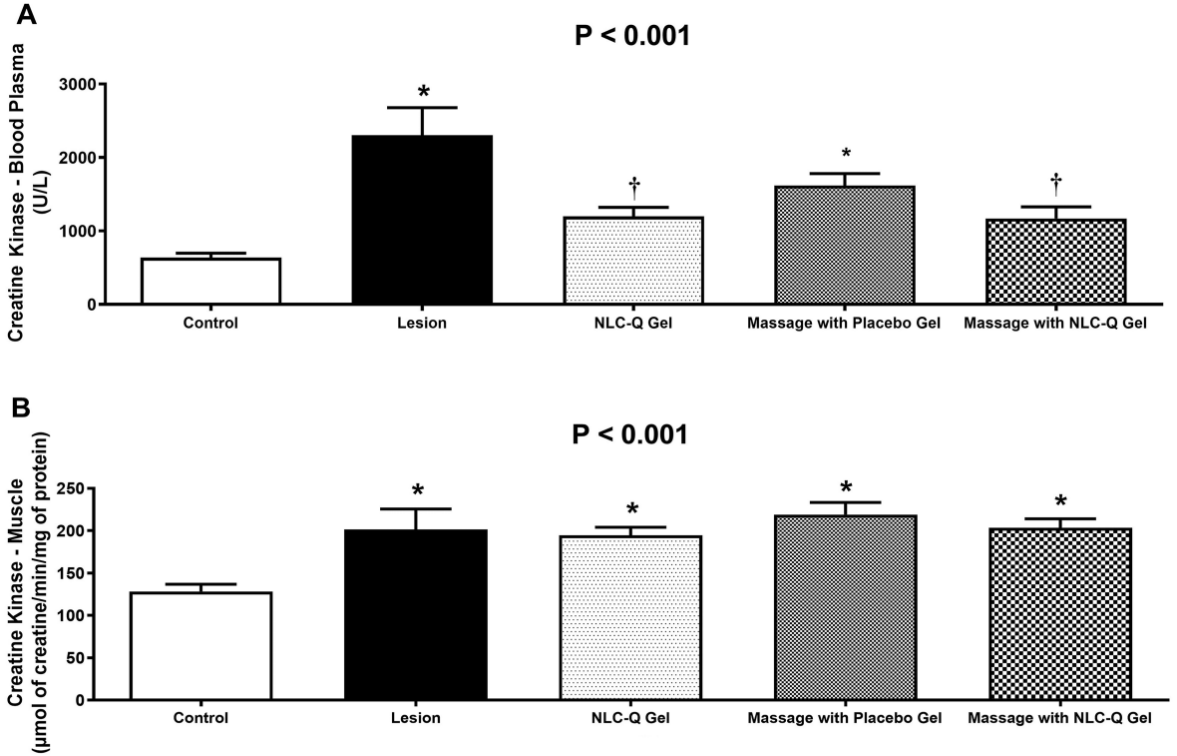
Results of creatine kinase enzymatic activity in blood plasma (**A**) and gastrocnemius muscle (**B**). Data are reported as means±SD. *P<0.05 *vs* Control Group; ^†^P<0.05 *vs* Lesion Group (ANOVA). NLC-Q: nanostructured lipid carriers with quercetin.

In the blood plasma (Figure 2A), CK concentrations increased by 267% in the Lesion Group compared to the Control Group (MD: 1703; 95%CI: 900 to 2505 U/L, P<0.001) and 136% in the Massage with Placebo Gel Group (MD: 870; 95%CI: 68 to 1672 U/L, P=0.027). The NLC-Q Gel (P=0.284) and Massage with NLC-Q Gel (P=0.333) groups showed results similar to those of the Control Group. Compared to the Lesion Group, the interventions reduced plasma CK by 49% in the NLC-Q Gel Group (MD: -1141; 95%CI: -338 to -1943 U/L; P=0.002), 36% in the Massage with Placebo Gel Group (MD: -832; 95%CI: -30 to -1943 U/L; P=0.039), and 50% in the Massage with NLC-Q Gel Group (MD: -1125; 95%CI: -307 to -1634 U/L; P=0.001). The intervention groups (NLC-Q Gel, Massage with Placebo Gel, and Massage with NLC-Q Gel) showed no differences.

In skeletal muscle (Figure 2B), CK activity increased by 57% in the Lesion Group (MD: 73; 95%CI: 14 to 132 µmol of creatine/min/mg of protein; P<0.01), 51% in the NLC-Q Gel Group (MD: 90; 95%CI: 30 to 150 µmol of creatine/min/mg of protein; P=0.021), 71% in the Massage with Placebo Gel Group (MD: 74; 95%CI: 15 to 134 µmol of creatine/min/mg of protein; P<0.001), and 58% in the Massage with NLC-Q Gel Group (MD: 74; 95%CI: 15 to 134 µmol of creatine/min/mg of protein; P=0.007). The interventions did not result in any differences in muscle CK activity.

### Oxidative damage markers

**Figure 3:**
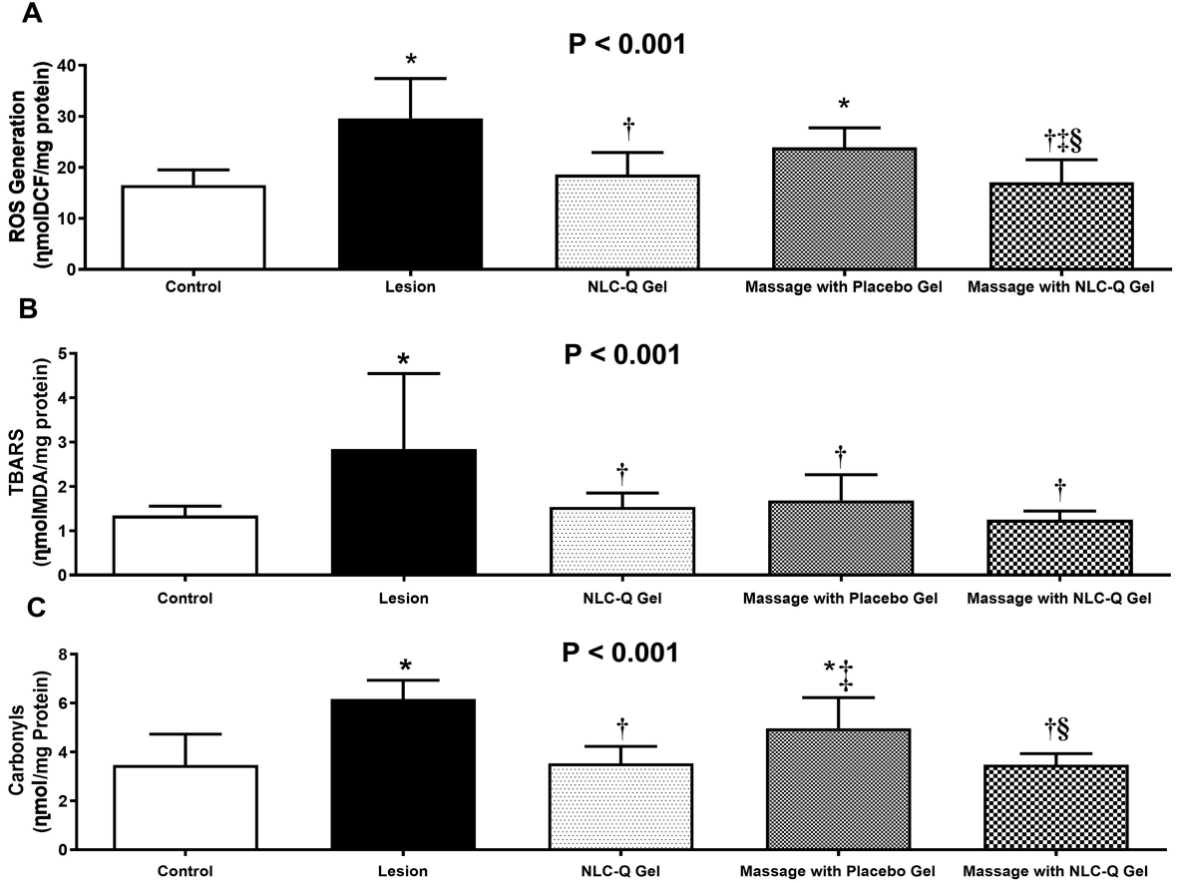
Effect of treatments on oxidative damage parameters in gastrocnemius muscle submitted to contusion lesion. Data are reported as means±SD. **A**, concentration of reactive oxygen species (ROS); **B**, TBARS: thiobarbituric acid reactive substances; **C**, protein oxidation. *P<0.05 *vs* Control Group; ^†^P<0.05 *vs* Lesion Group; ^‡^P<0.05 *vs* NLC-Q gel group; ^§^P<0.05 *vs* Massage with Placebo Gel Group (ANOVA). NLC-Q: nanostructured lipid carriers with quercetin.

Oxidative damage was assessed by measuring total ROS generation (H_2_DCF-DA), lipid peroxidation (TBARS), and protein oxidation (carbonyl). The data are reported in Figure 3.

ROS production increased 78% (MD: 13.1; 95%CI: 6.3 to 19.8 ղmolDCF/mg protein; P<0.001) in the Lesion Group and 44% (MD: 7.35; 95%CI: 0.8 to 13.9 ղmolDCF/mg protein; P=0.021) in the Placebo Massage Group compared to the Control Group (Figure 3A). The NLC-Q Gel (P=0.903) and Massage with NLC-Q Gel (P=0.980) groups did not differ from the Control Group. The Massage with Placebo Gel Group showed equivalent results to those of the Lesion Group (P=0.133). However, ROS decreased by 38% in the NLC-Q Gel Group (MD: -11; 95%CI: -4.3 to -17.8 ղmolDCF/mg protein, P<0.001) and by 43% in the Massage with NLC-Q Gel Group (MD: -12.5; 95%CI: -5.7 to -19.3 ղmolDCF/mg protein, P<0.001). ROS generation decreased by 29% (MD: -6.8; 95%CI: -0.2 to -13.8 ղmolDCF/mg protein, P=0.037) in the Massage with NLC-Q Gel Group compared to the Massage with Placebo Gel Group.

Lipid peroxidation (Figure 3B) increased by 212% in the Lesion Group (MD: 1.5; 95%CI: 0.4 to 2.6 ղmolMDA/mg protein; P=0.003) compared to the Control Group. The NLC-Q Gel (P=0.987), Placebo Massage (P=0.903), and Massage with NLC-Q Gel (P=0.998) groups did not differ from the Control Group. Compared to the Lesion Group, a reduction of 46% was observed in the NLC-Q Gel Group (MD: -1.3; 95%CI: -0.2 to -2.4 ղmolMDA/mg protein; P=0.014), 40% in the Massage with Placebo Gel Group (MD: -1.2; 95%CI: -0.05 to -2.3 ղmolMDA/mg protein; P=0.037), and 56% in the Massage with NLC-Q Gel Group (MD: -1.5; 95%CI: -0.5 to -2.7 ղmolMDA/mg protein; P=0.002). The intervention groups showed no differences in lipid peroxidation.

Protein oxidation (carbonyls) increased by 78% (MD: 2.7; 95%CI: 1.3 to 4.1 ղmol/mg Protein; P<0.001) and 41% in the Massage with Placebo Gel Group (MD: 1.5; 95%CI: 0.1 to 2.9 ղmol/mg Protein; P=0.014) compared to the Control Group. The results for the NLC-Q Gel (P=0.998) and Massage with NLC-Q Gel (P=0.999) groups were similar to those of the Control Group. Regarding the Lesion Group, protein oxidation decreased by 36% in the NLC-Q Gel Group (MD: -2.2 95%CI: -0.8 to -3.6 ղmol/mg Protein; P<0.001) and 43% in the Massage with NLC-Q Gel Group (MD: -2.7; 95%CI: -1.2 to -4.1 ղmol/mg Protein; P<0.001). Protein oxidation decreased by 29% in the NLC-Q Gel Group (MD: -1.4; 95%CI: -0.2 to -2.7 ղmol/mg Protein; P=0.012) and 30% in the Massage with NLC-Q Gel Group (MD: -1.5; 95%CI: -0.2 to -2.7 ղmol/mg Protein; P=0.015) compared with the Massage with Placebo Gel Group (Figure 3C).

### Antioxidant analysis

**Figure 4:**
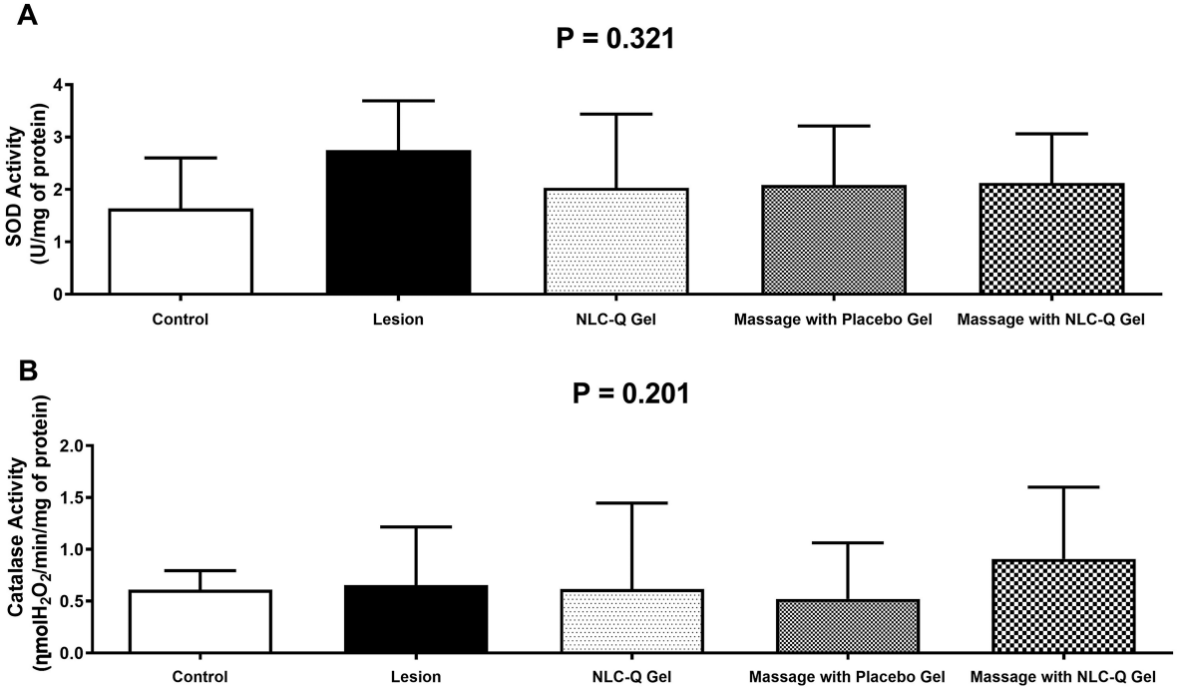
Effect of treatments on antioxidant enzyme activities in gastrocnemius muscle submitted to contusion lesion. Data are reported as means±SD (ANOVA). **A**, Superoxide dismutase (SOD); **B**, catalase. NLC-Q: nanostructured lipid carriers with quercetin.

The data for SOD and CAT activities are reported in Figure 4. The enzymatic SOD (Figure 4A; P=0.469) and CAT (Figure 4B; P=0.204) activities did not differ between groups.

## Discussion

Muscle injury caused by contusion induced OS, as demonstrated by increases in total ROS generation and oxidation of lipids and proteins. The lesion also provoked an inflammatory response, as indicated by increased muscle CK activity and blood plasma concentrations of this enzyme. These results align with those of previous studies that used this model (6,8,9,28).

The interventions applied 24 h after injury decreased CK concentrations in blood plasma but did not alter the activity of this enzyme in the injured muscle. Increased muscle CK activity is expected after contusion muscle injury (6,9). However, the inflammatory phase of muscle damage can be measured by increased CK a few hours after injury (6,7,29). OS causes alterations in the structure of muscle fibers, disrupting the sarcolemma and extravasation of specific metabolic enzymes from the sarcoplasm and mitochondria (30). This mechanism, characterized by lower extravasation of CK into the blood, suggests that the interventions led to a reduction in injury. Previous studies in humans have shown that massage reduces plasma CK concentrations (18,23,31), which is associated with a decrease in delayed-onset muscle soreness (18,31). The NLC-Q Gel also reduces CK in the blood plasma (9), and the joint actions of these therapeutic methods favor this effect.

The activity of the antioxidant enzymes (SOD and CAT) remained unchanged during the experiment. Similar results of SOD (6) and CAT (9,29) have been previously described in the literature, showing that after 5 days of muscle injury caused by this contusion model, these enzymes practically return to their regular activity (8). However, increases in total ROS generation and lipid and protein oxidation indicated oxidative damage, which is in line with previous studies (6,8,9). These results suggest that the favorable effects of the interventions (NLC-Q Gel and Massage with NLC-Q Gel) on measures of oxidative damage (total ROS generation and protein) did not occur because of the improvement in the enzymatic antioxidant capacity of SOD and CAT, but rather because of the antioxidant capacity of quercetin (9,1214,).

In this study, NLC-Q Gel, Massage with Placebo Gel, and Massage with NLC-Q Gel reduced lipoperoxidation. The reduction in lipoperoxidation by massage (19) and NLC-Q gel (9) was consistent with previous findings. However, only the NLC-Q Gel and the Massage with NLC-Q Gel decreased total ROS generation and protein oxidation. These results suggest that quercetin has antioxidant and anti-inflammatory properties (12,14), which have already been demonstrated with quercetin gel complexed with β-cyclodextrin (28). Notably, this study used nanostructured lipid carriers, which favor the topical administration of drugs because they can support greater drug loading, retention efficiency, and physical stability during storage, increase apparent solubility, control release rate, and improve bioavailability of encapsulated lipophilic compounds compared with other carriers (9,32). This penetration is facilitated by micro-lesions from abrasion to the stratum corneum (33) caused by the depilation of the animals in the present study.

The application of NLC-Q Gel and Massage with NLC-Q Gel showed better effects than Massage with Placebo Gel, as these interventions reduced the total generation of ROS and oxidation of proteins (carbonyls). Although massage is widely used for recovery of exercise and sports injuries (18,31), the neurophysiological mechanisms underlying this practice remain poorly understood and require further clinical evidence (18). However, in this study, this therapy reduced blood CK concentrations and lipid damage. The NLC-Q Gel exhibited additional effects, as it attenuated the total generation of ROS and protein oxidation. This is because of the antioxidant and anti-inflammatory actions of this polyphenol (12,14).

This study demonstrated that Massage with NLC-Q Gel does not have any additional effects compared to NLC-Q Gel alone. This may have occurred because of the increased penetration of quercetin contained in the NLC-Q Gel through the stratum corneum, which was facilitated by skin abrasion caused by depilating the animals (33). Among the limitations of this study are the absence of histological evaluations of injured muscle tissue, glutathione measurements (GSH/GSSG), inflammatory markers (TNF-α, IL-1β, and IL-6), and quercetin concentrations in blood plasma. These measures would strengthen this study's findings.

In conclusion, in an experimental model of muscle contusion in rats, NLC-Q Gel, Massage with Placebo Gel, and Massage with NLC-Q Gel applied 24 h after injury reduced CK concentrations in blood plasma and attenuated lipid peroxidation. However, only the NLC-Q Gel and Massage with NLC-Q Gel reduced total ROS generation and protein oxidation (carbonyls). Although the NLC-Q gel can be applied during massage, the massage itself showed no additional effects. However, the results of the NLC-Q Gel are promising, and the interactions with massage should be clinically assessed to determine their antioxidant and anti-inflammatory effects on skeletal muscle injury in humans.

## Data Availability

All data generated or analyzed during this study are included in this published article.
